# Bioaerosols in swine confinement buildings: A metaproteomic view

**DOI:** 10.1111/1758-2229.13208

**Published:** 2023-11-02

**Authors:** Susann Meyer, Nicole Hüttig, Marianne Zenk, Udo Jäckel, Dierk‐Christoph Pöther

**Affiliations:** ^1^ Federal Institute for Occupational Safety and Health Berlin Germany; ^2^ Research Institute for Farm Animal Biology (FBN) Dummerstorf Germany

## Abstract

Swine confinement buildings represent workplaces with high biological air pollution. It is suspected that individual components of inhalable air are causatives of chronic respiratory disease that are regularly detected among workers. In order to understand the relationship between exposure and stress, it is necessary to study the components of bioaerosols in more detail. For this purpose, bioaerosols from pig barns were collected on quartz filters and analysed via a combinatorial approach of 16S rRNA amplicon sequencing and metaproteomics. The study reveals the presence of peptides from pigs, their feed and microorganisms. The proportion of fungal peptides detected is considered to be underrepresented compared to bacterial peptides. In addition, the metaproteomic workflow enabled functional predictions about the discovered peptides. Housekeeping proteins were found in particular, but also evidence for the presence of bacterial virulence factors (e.g., serralysin‐like metalloprotease) as well as plant (e.g., chitinase) and fungal allergens (e.g., alt a10). Metaproteomic analyses can thus be used to identify factors that may be relevant to the health of pig farmers. Accordingly, such studies could be used in the future to assess the adverse health potential of an occupationally relevant bioaerosol and help consider defined protective strategies for workers.

## Graphical abstract

This study uses metaproteomic analyses to identify factors in the air of the barn that may be relevant to the health of pig farmers. The study reveals the presence of airborne peptides from pigs, their feed and different (potentially pathogenic) microorganisms, and allows functional predictions to be made about the peptides detected. Mainly housekeeping proteins were found, but also evidence for the presence of bacterial virulence factors and plant and fungal allergens.

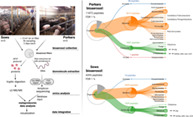

## INTRODUCTION

Exposure to bioaerosols in intensive livestock buildings are intensively discussed as risks for animals' as well as workers' health (Cormier et al., 2000; Danuser, 2000; Douglas et al., 2018; Hall et al., 2020; Chmielowiec‐Korzeniowska et al., 2021; Tang et al. 2021). Microorganisms (bacteria, archaea, fungi and viruses), plants, their biomolecules (lipids, DNA, Lipopolysaccharides (LPS, endotoxins) and proteins) and their spores and pollen have already been identified in bioaerosols, which inevitably leads to poor air quality in high concentrations (Fröhlich‐Nowoisky et al., 2016). The underlying microbial composition and concentration of bioaerosols in intensive livestock workplaces varies strongly and is dependent on animal type, season, feed and production type (Duchaine et al., 2000; Hong et al., 2012; Kumari & Choi, 2014; Létourneau et al., 2009; Nehmé et al., 2008; Tang et al., 2020). However, defined organism species are always encountered in the respective workplaces. In pig barns, the main bioaerosol contamination comes from pig manure slurry, which contains the faecal microbiome of the pigs (Nehmé et al., 2008; Yan et al., 2019). Therefore, Lactobacilli and Clostridia, among others, are widely distributed in the bioaerosol of commercial pig farming and can be used as indicator microorganisms for this type of farming (Clauß, 2020; Gärtner et al., 2016).

To identify the diversity of airborne microorganisms, on one hand, studies are based on cultivation techniques (Fallschissel et al., 2010; Martin et al., 2009), which provide an overview of culturable microorganisms within the range of the given growth conditions. On the other hand, genomic analyses such as PCR methods, whole metagenomic and amplicon sequencing analyses are performed (Bonifait et al., 2014; Fallschissel et al., 2010; Martin et al., 2009; Yan et al., 2019), leading to the closest approximation of the real taxonomic diversity and, in the best case, functional potential of microbial communities. However, with the exception of the whole metagenomic approach, these approaches lack taxonomic depth down to the species level. Thus, most of these methods alone do not cover the true composition of microorganisms or do not distinguish between metabolically active and non‐active cells (Kleiner, 2019; Yan et al., 2019).

Metaproteomics can provide new insights into bioaerosols by analysing the expressed proteins of airborne organisms (Kleiner, 2019) and could answer questions as to whether physiologically active microorganisms and their virulent or allergenic products are present in bioaerosols for triggering infections and allergies or acting toxic on workers' health. Nevertheless, the analysis of metaproteomes from environmental air samples is still a challenge. Thus, only a few studies have analysed the metaproteome and defined proteomic biomarkers within bioaerosols by mass spectrometric techniques so far (Druckenmüller et al., 2017; Liu et al., 2016; Piovesana et al., 2019). That is why the aim of this study was to apply a universal metaproteomic workflow for the detection of proteins from bioaerosol samples and, from an occupational safety and health perspective, to focus on the detection of proteins that could have infectious, sensitizing, or toxic effects on workers in swine barns.

## EXPERIMENTAL PROCEDURES

### Characteristics of swine confinement buildings (SCBs)

Two different types of SCBs were available at the experimental pig facility of the Research Institute for Farm Animal Biology (FBN, Dummerstorf). The selected sow barn contained 31 gestating sows (up to 3 years old, giving birth several consecutive times) in an approximately room volume of 820 m^3^, whereas the porker barn (breeding pigs, from 70 up to 125 day of life) was approximately 310 m^3^ and housed 32–46 porkers. The porkers were given straw as an occupational material and the sows were also given a bedding mixture of chopped straw, sawdust and spelt litter. The pigs were kept on slats through which the pig secretions drained off and were collected in collection tanks. In case of overfilling, these troughs were emptied by opening the supply lines for collection tanks and allowing the slurry to drain underground. In general, the barns were cleaned twice a day by the employees.

### Bioaerosol collection

Bioaerosol samples were collected from 20 August 2019 to 23 August 2019 in the barns for 3 days each. Both barns had similar average temperatures between 21°C and 24°C and average relative humidity of 57%–70% (Table 1) as determined by a multimeter (BAPPU‐classic, ELK GmbH, Krefeld, Germany). The barns were cleaned once during sampling on the first and the third day and they were cleaned twice during the second sampling day. In each case, collections did not occur during or immediately after cleaning. Collections were made 1.50 m above the ground well away from ventilation, windows and doors. Quartz filter (Labscience, #QFH 076, Ø = 76 mm) were prepared by UV‐light incubation as well as heat sterilization (240°C) for 1 h and stored sterile packed. Sterile polycarbonate filters (Whatman, #110809, Ø = 37 mm) were stored in already sterilized filter holders. Bioaerosols were then collected on both filter types for subsequent total cell counting and proteomic studies. The quartz filters were exposed to bioaerosols using stationary sampling systems (MP2/39 and Gallus 2000, Umweltanalytik Holbach GmbH, Wadern, Germany) with an air flow of maximal 39 L/min to collect total dust samples, and the polycarbonate filters were exposed using sampling systems (SG10‐2, DEHA Haan and Wittmer GmbH, Heimsheim, Germany) with an air flow of 10 L/min to collect inhalable particles. The overall volume of air loaded on the filters are presented in Table 1. Additionally, control filters were put in the cleaned sampling systems without an airflow. All filters were stored in sterilized aluminium foil at −80°C before usage.

**TABLE 1:** Sampling system and air conditions in the barns during bioaerosol sampling.

Barn	Day	Sampling system	Air volume (m^3^)	Air flow (L/min)	Sampling time (min)	Average temperature θ (°c)	Average relative air humidity ϕ (%)	Average flow velocity ω (m/s)
SOWS	1	MP2/39	12	36	332	24.1	53.2	0.08
		SG10‐2	3.3	10	334			
PORKERS		MP2/39	12	35	339	24.4	53.7	0.09
		SG10‐2	3.4	10	341			
SOWS	2	MP2/39	14	36	385	21.6	64.1	0.14
		SG10‐2	3.9	10	387			
PORKERS		MP2/39	14	36	384	22.8	60.4	0.11
		SG10‐2	3.9	10	389			
SOWS	3	MP2/39	13	39	335	21.7	61.6	0.10
		SG10‐2	3.5	10	351			
PORKERS		MP2/39	12	34	349	23.2	56.3	0.29
		SG10‐2	3.5	10	347			

### Bioaerosol sample preparation

The extraction of cells from polycarbonate filters were carried out by paddle blending the filters (60s, Stomacher®80 micro‐Biomaster, Seward Limited, Worthing, UK) in 10 mL of physiological sodium chloride solution (0.9% (w/v) NaCl). The extracted cells were treated with formaldehyde solution (37% (v/v) and labelled with DAPI (4′,6‐diamidino‐2‐phenylindole). Finally, total cell counting of all samples were applied by a defined microscopic method (Klug et al., 2010a, 2010b; Klug & Jäckel, 2012; VDI 4253 Part 4, 2013).

The quartz filters (Ø 76 mm ≙ 45,36 cm^2^) were divided into two parts. Approximately 8 cm^2^ were used for DNA extraction and 37 cm^2^ for protein extraction. DNA was extracted as described in Laufer et al. (2021). In brief, the filter parts were incubated with 900 μL 0.5 mg/mL Proteinase K for 30 min at 50°C with 1000 rpm. Additionally, 0.5 mL Zirconia/Glass‐Beads (0.1 mm, Fa. Carl Roth, Karlsruhe, Germany) were added and DNA was extracted using the Plant Genomic DNA extraction kit (Sigma Aldrich). DNA content was determined using the QuantiFluor ONE dsDNA‐System in a Quantus Fluorometer (both Promega).

The extraction of proteins from quartz filters were carried out by paddle blending the filters (60s, Stomacher®80 micro‐Biomaster, Seward Limited, Worthing, UK) with 8 mL of 5% (w/v) SDC (sodium deoxycholate) solubilized in 50 mM Tris–HCl (pH 8.0) in sterile plastic bags and heated at 90°C for 15 min (conditions were tested in previous work) with a subsequent sonication step for 3 min. After centrifugation, the supernatants were stored at −20°C for at least 1 h. Purification and digestion of proteins based on the adapted single pot solid‐phase enhanced sample preparation (SP3*) protocol published by Blankenburg et al. (2019) with following changes. We used hydrophobic and hydrophilic magnetic beads from GE Healthcare (Sera‐Mag SpeedBeads™ Carboxyl‐Magnet‐Beads, hydrophob; Sera‐Mag SpeedBeads™ Carboxyl‐Magnet‐Beads, hydrophile), mixed 10 μL of beads (50:50) and prepared them freshly by strongly washing with 50 μL ultra‐pure water five times. The beads mix was suspended in 25 μL ultra‐pure water and 2 μL of this mix was added to each of the protein samples. A final concentration of 70% (v/v) acetonitrile (ACN) was adjusted before shaking (INFORS HT Ecotron, INFORS AG, Bottmingen, Switzerland) for 20 min at 160 rpm. The beads were then separated and strongly washed twice with 1 mL 100% (v/v) Ethanol and 1 mL 100% (v/v) ACN in a Magna‐50 mL‐Rack by sedimentation for 2 min, before they were air‐dried. Protein samples were digested with 200 ng trypsin in 20 mM TEAB (Triethylammonium bicarbonate buffer) solution (18 h, 37°C) and peptides were eluted with 12.5 μL 2% DMSO (Dimethyl sulfoxide) and incubated in an ultrasonic bath for 3 min. Samples were then purified with Pierce™ C18 tips according to the protocol of the manufacturer using the following solutions: Wetting solution: 50:50 ACN (Acetonitrile):water; Equilibration solution: 0.1% FA (Formic acid) in water; Rinse solution: 0.1% FA in 5% ACN:water; Elution with 10 μL 0.1% FA in 60% ACN:water. After removing acetonitrile by a vacuum centrifuge step and making up the sample to 10 μL with 0.1% FA in water, each sample was analysed twice by LC–MS/MS.

### Metagenomic data analysis

Amplicon sequencing was carried out at LGC (Biosearch Technologies, Berlin, Germany) as followed: For determining the fungal composition primers ITS4 (5′‐TCCTCCGCTTATTGATATGC‐3′) and fITS7 (5′‐GTGARTCATCGAATCTTTG‐3′) targeting variable ITS2 regions of Fungi and for the Prokaryotes composition the Klindworth‐Primer‐Set targeting 341F (5′‐CCTACGGGNGGCWGCAG‐3′) and 785R (5′‐GACTACHVGGGTATCTAATCC‐3′) region of the 16S rRNA gene were utilized (Ihrmark et al., 2012; Klindworth et al., 2013; White et al., 1990). Samples were analysed using Illumina MiSeq V3 300 bp paired end with app. 200,000 reads per sample. Sequences were demultiplexed utilizing Illumina bcl2fastq v2.20 software, allowing 1 or 2 mismatches or Ns in the barcode if possible and subsequently sorted by amplicon inline barcodes and barcodes were clipped. Afterwards, reads with less than 100 bp were discarded and primers were clipped allowing 3 mismatches per primer. If necessary, sequences were turned into forward‐reverse‐primer orientation. After preprocessing at LGC sequences were subject to data analysis employing Qiime 2 2021.4 (Bolyen et al., 2019) by importing the paired end sequences using the input format PairedEndFastqManifestPhredd33V2. Denoising was carried out using DADA2 (Callahan et al., 2016). Taxonomy assignments to amplicon sequence variants were carried out by “feature classifier classify sklearn” with a naïve Bayes trained taxonomy classifier. For fungal annotation the Unite 8.3 database was imported as classifier into QIIME2 (Abarenkov et al., 2010, 2021; Quast et al., 2012), for bacterial annotation the SILVA 132 database was imported into QIIME2 by the RESCRIPt plugin (Quast et al., 2012; Robeson et al., 2021; Yilmaz et al., 2014). For visualization purposes the “level 7″ information of the taxabarplots were exported into a csv‐file which was converted to be readable for Krona Tools (Ondov et al., 2011). Amplicon sequencing data were deposited to the NCBI BioProject Database (https://www.ncbi.nlm.nih.gov/bioproject/) with accession number PRJNA1004577.

### Metaproteomic data analysis

Peptide samples were analysed by an Ultimate 3000 coupled to a QExactive Plus (Thermo Scientific, Bremen, Germany). After loading the peptides onto the analytical column with buffer A (water in 0.1% formic acid) (Acclaim PepMap RSLC C18, 2 μm, 100 Å, 75 μm ID × 25 cm) a binary 115 min gradient from 5% to 95% buffer B (80:20 acetonitrile in 0.1% formic acid: water in 0.1% formic acid) at a flow rate of 300 nL/min was used to separate the peptides from bioaerosol samples. Precursor spectra were acquired in profile mode in the scan range of 350–1600 m/z with a resolution of 70,000, an automatic gain control (AGC) of 1 × 10^6^ and maximum injection time of 100 ms. A data dependent acquisition (DDA) of the 10 most intense precursor ions was performed by collision‐induced dissociation with a normalized collision energy of 27.5%, with an AGC of 2 × 10^5^ and maximum injection time of 100 ms. The isolation window was set to 2 m/z. Dynamic exclusion was set to 30 s and ions with unknown, single or charges >7 were excluded from fragmentation. The fragment ions were detected at 17,500 resolution with a fixed first mass of 200 m/z.

The acquired raw data per condition were processed in the Proteome Discoverer (PD, version 2.5.0.400, Thermo Scientific). The recalibration of the MS/MS spectra was performed via the implemented PD node “Spectrum Files RC” with default parameter. The database searches of acquired LC–MS/MS spectral data were performed using the 2stage Sequest HT algorithm with subsequent intensity‐based INFERYS rescoring of Sequest HT search engine results (Eng et al., 1994; Gessulat et al., 2019; Zolg et al., 2021). Due to the high diversity and differences of bacterial and fungal families in the selected barns, we decided to create separate defined databases for sow and porker barns respectively. Each protein database contained sequences downloaded from UniProtKB/TrEMBL (03/2021) belonging to bacterial and fungal families identified by metagenomic analysis with at least 50 sequence reads. In addition, all protein sequence entries from UniProtKB/SwissProt database (03/2021) were combined to both databases. Redundant sequences were filtered and headers shortened. Finally, a database of 31,521,021 nonredundant protein sequence entries specific to sow bioaerosol and a database of 18,127,275 nonredundant protein sequence entries specific to porker bioaerosol were used for the Sequest HT database searches. The overlap between both databases are 15,091,821 protein sequences.

In both phases of the database search, the following parameters were used: usage of databases described above, 0.02 Da fragment ion mass tolerance, 10 ppm parent ion tolerance, maximum two missed cleavages, full tryptic digestion, default modification per search step, FDR of 0.01 on peptide level by rescoring the spectra with Percolator (Käll et al., 2007; The et al., 2016). After adhering to a strict peptide FDR of 0.01, we accepted all peptide identifications, including those matching only by a single peptide spectrum (PSM).

The metaproteomic software Unipept 4.5.0 was used for peptide alignment to complete UniProt 2020.01 by the lowest common ancestor (LCA) algorithm. The following settings were selected: equate isoleucine and leucine when matching the peptides to UniProt entries, filter out duplicate peptides, use advanced missed cleavage handling (Gurdeep Singh et al., 2019; Mesuere et al., 2015, 2016). The mass spectrometry proteomics data have been deposited to the ProteomeXchange Consortium via the PRIDE (Perez‐Riverol et al., 2022) partner repository and with the dataset identifier PXD039685. To visualize the taxonomic annotations the tree views and complete data sets were exported from unipept (https://unipept.ugent.be/datasets) and Krona‐plots were generated (Ondov et al., 2011). Functional analyses were supported by unipept based on the assignment of peptides to GO terms (The Gene Ontology Consortium, 2019), InterPro entries (Blum et al., 2021), and EC (Enzyme Commission) numbers. Sankey diagrams (https://app.rawgraphs.io/) were created to combine the taxonomic and functional annotations for which at least 10 peptides were found (Mauri et al., 2017).

## RESULTS AND DISCUSSION

### Total cell counts

**FIGURE 1:**
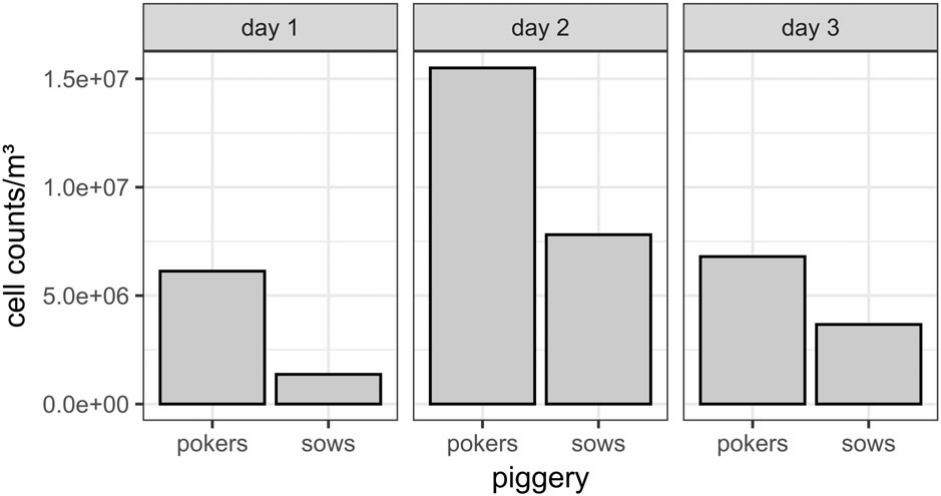
Amounts of cells per m^3^ inhalable air in porkers barn and sows barn at 3 different days.

Comparing the three sampling days of each pig barn, the determined amounts of microbial cells per m^3^ air varied between 2 × 10^6^ and 1.5 × 10^7^ cells (Figure 1). These cell counts were in the same order of magnitude as the already published data from commercial pig farming (Bonifait et al., 2014; Gärtner et al., 2016). In detail, our data pointed out that the amount of detected cells were consistently higher in porker barn than in the sow barn on the 3 days of testing (Figure 1). The conditions in the two barns did not vary much per day in terms of temperature, humidity and air flow (Table 1). However, the occupancy of the pigs in the two barns varies, so that the porkers barn with only 310 m^3^ houses 32–46 animals, while the sow barn has 31 sows distributed in 820 m^3^. We therefore suspect that microbial contamination of the air increases when more animals are housed in a smaller space. In addition, the number of cells per m^3^ of air in both barns was lower on the first and third days than on the second day. We explain the lower cell counts observed on both days partly by the fact that the barns were cleaned only once during bioaerosol sampling. In comparison, cleaning was performed twice on the second day of sampling during bioaerosol collection and an increased number of cells was detected. This could be an indication that during the times when the barns are cleaned, the amount of airborne cells and particles increases leading to an increased bioaerosol exposure for employees. This could also be described for the keeping of horses (Grzyb et al., 2022).

### Meta‐omics data

Amplicon sequencing of the 16S rRNA genes of Prokaryotes and the ITS2 gene regions of Fungi were performed to identify microorganisms in the selected pig barns. Our data showed that all three replicates were quite similar with respect to the occurrence of similar taxa and lead to reproducible data (Supporting Information 1). They reflected bacterial composition in pig barns air already analysed by molecular biological methods (Clauß, 2020; Gärtner et al., 2016).

However, amplicon sequencing is associated with limitations for a quantitative analysis of microbial composition, as already described in Gärtner et al. (2016). For example, it should be noted that the 16S rRNA gene, and in particular the ITS region may not be present only once in a genome, but may occur in different numbers or have different amplification efficiencies depending on the organism and environmental changes (Lavrinienko et al., 2021; Stoddard et al., 2015).

However, the genomic identifications from amplicon sequencing allowed us to create specific reference protein databases of the selected pig barns for the metaproteomic analyses. The metaproteomic analyses revealed strong taxonomic similarity of our 3‐day sample results per barn (Supporting Information 1), so we decided to perform 3 days‐combined database searches per barn (*n* = 3, FDR = 1%). Accordingly, a total of 11,872 different peptides from the porker barn and 4395 different peptides from the sow barn could be assigned by Unipept. We therefore assume a generally higher load of airborne microorganisms in the porker barn compared to the sow barn based on the amount of different identified peptides, which is also supported by the total cell counts/m^3^ per barn.

**FIGURE 2:**
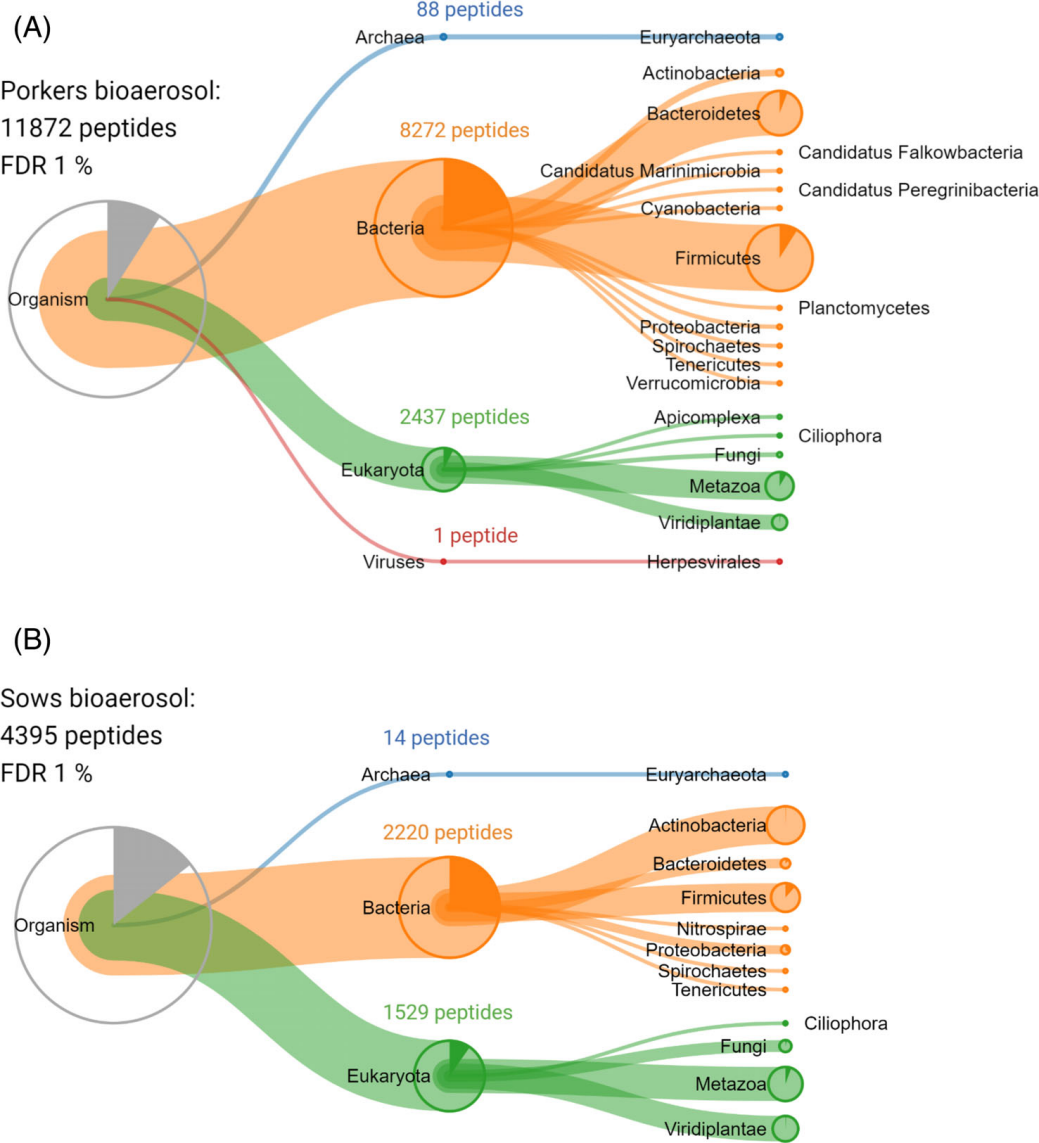
Taxonomic distribution of identified peptides from porkers (A) and sows (B) barn.

The complete taxonomic data of both barns are presented in Supporting Information 2. Briefly, peptides in both barns were assigned to the superkingdoms of Eukaryota, Bacteria and Archaea. In addition, some peptides are highly conserved among all organisms (Figure 2; grey fractions in the pie chart at “organism” level).

Most peptides from Eukaryota could mainly be assigned to the phyla Viridiplantae and Metazoa. In detail, both data sets indicate that most of the peptides from Eukaryota are unique to the family of Suidae (pigs) and its feed components (plants) (Supporting Information 2). It should still be mentioned here that among plant proteins there may be some allergens that are harmful to human health (Sinha et al., 2014; Taketomi et al., 2006; Żukiewicz‐Sobczak et al., 2013). Usually, these molecules are known to cause allergenic reactions such as external skin irritation, rhinitis, and bronchial asthma due to reactivity with immunoglobulin E (IgE) antibodies in people who have sensitization after exposure (Sinha et al., 2014; Taketomi et al., 2006). These allergens include, for example, the food allergens, chitinase, thioredoxin, gliadin and trypsin inhibitor (serpin) from sweet grasses as barley and wheat (Fasoli et al., 2009; Palosuo, 2003; Volpicella et al., 2017), which we were identified in the pig barns bioaerosols (Supporting Information 2). We assume that the concentration of these allergens in the air around the employees is more enriched especially during the corresponding work tasks involving feeding of the pigs and cleaning of the barns.

**FIGURE 3:**
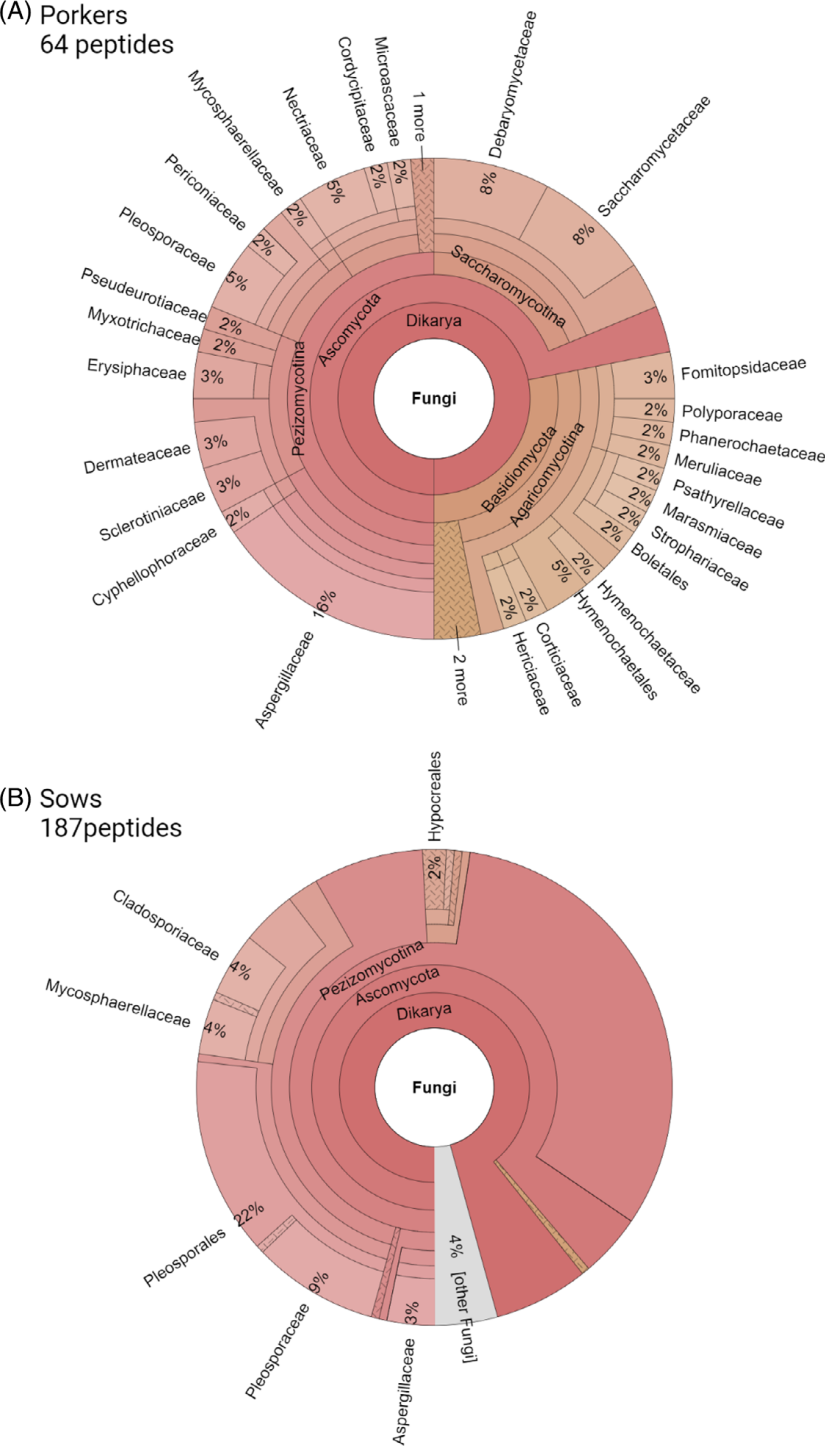
Taxonomic distribution of identified fungal peptides from porkers (A) and sows (B) barns.

In addition, low amounts of peptides could be assigned to the phyla Fungi as well as Apicomplexa (eukaryotic parasites) and Ciliophora (ciliates). In case of Fungi, 187 peptides could be found in sow and only 64 peptides in porker bioaerosols. Figure 3 shows that mainly peptides from the fungal families Aspergillaceae, Debaryomycetaceae, Saccharomycetaceae and Pleosporaceae can be identified in porker bioaerosol. Detected peptides in sow barn bioaerosol, indicate that Pleosporaceae, Mycosphaerellaceae and Cladosporiaceae are the most common fungal families. Harmful effects of fungi result from mould infection or exposure to mycotoxins, microbial volatile organic compounds (MVOCs) or some intracellular housekeeping proteins such as proteases and enzymes. The latter can act as allergens and trigger a range of allergic reactions of the respiratory tract and the skin (Pagano et al., 2011; Simon‐Nobbe et al., 2008; Żukiewicz‐Sobczak et al., 2013). In this study, we have now been able to find evidence for peptides in bioaerosols that correspond to mould allergens, for instance alt a7 (flavodoxin) and alt a10 (aldehyde dehydrogenase) from *Alternaria* as well as the as Cla h6 (enolase) from *Cladosporium herbarium* (Simon‐Nobbe et al., 2008). Nevertheless, we assume that moulds are not dominant in our samples, since we found only a small number of specific fungal peptides. This has already been noted in other studies examining culturable bacteria and fungi in bioaerosols from pig barns with conventional livestock farming (Yang et al., 2022). In general, it can be assumed that keeping pigs on slats, without straw bedding on the ground, the fungal load plays a rather minor role in bioaerosols.

In case of identified Prokaryotes the identified Archaea peptides (porkers: 88 peptides, sows: 14 peptides) mainly assigned to the genus of *Methanobrevibacter* being the most abundant archaeal genus in the intestines of pigs as well as in the human gut (Deng et al., 2021; Lurie‐Weinberger & Gophna, 2015; Mi et al., 2019; Oxley et al., 2010). It could already be isolated as a dominant archaeal genus from pig faeces (Gierse et al., 2020; Seshadri et al., 2018) and was previously described to be detectable in bioaerosols from pig farming (Kristiansen et al., 2012; Nehmé et al., 2009). A recent study by Barnett and colleagues now suggests that archaea, specifically *Methanobrevibacter* species (*M. stadtmanae* and *M. smithii*), may play a role in mitigating childhood asthma or allergy risk (Barnett et al., 2019).

In case of Bacteria, previous studies presented mainly Bacteriodetes, Firmicutes and Actinobacteria identified in swine confinement buildings by cultivation, 16S‐rRNA gene‐ and FISH analyses (Gärtner et al., 2016; Kristiansen et al., 2012; Wu et al., 2020). Our study here provides similar results but at the proteomic level. Furthermore, our study allows us to observe a different bioaerosol composition between the two barns. Accordingly, our analysis revealed Actinobacteria (35% unique peptides of all identified bacterial peptides) followed by Firmicutes (26% unique peptides of all identified bacterial peptides) in the sow barn and Firmicutes (47% unique peptides of all identified bacterial peptides) followed by Bacteriodetes (30% unique peptides of all identified bacterial peptides) in the air of porker barn.

On closer inspection of the sow barn, the high relative proportion of Actinobacteria are of particular importance. Their spores, like mould spores, can become airborne and cause respiratory health problems after exposure (UBA mould guide, 2017). Apart from that, it is known that some Actinobacteria are able to produce antibiotics and toxins (Su et al., 2015). Therefore, the Federal Environment Agency (UBA) in Germany certainly takes high concentrations of Actinobacteria in the indoor environment into account when assessing the indoor air quality (UBA mould guide, 2017).

**FIGURE 4:**
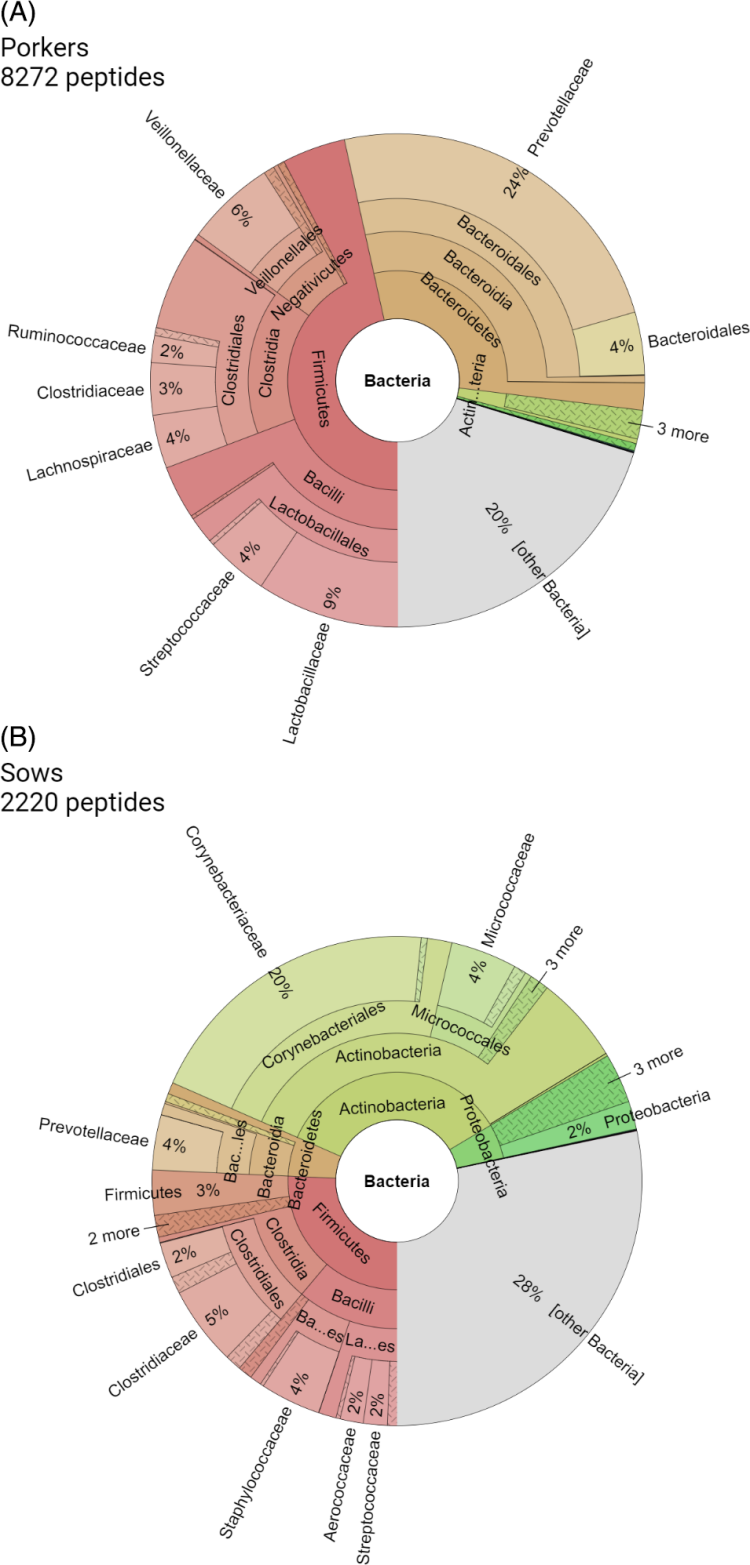
Taxonomic distribution of identified bacterial peptides from porkers (A) and sows (B) barns.

In the sow barn the dominant unique peptides with 20% of all detected bacterial peptides are those which could be assigned to the family Corynebacteriaceae (Figure 4) with 30% of them assigned to the species *Corynebacterium xerosis* (Supporting Information 2). Vela and Coworker have been able to detect the commensal organism *C. xerosis* in joints of pigs with arthritis, as well as in different organs of pigs with subcutaneous abscesses or respiratory problems, and also from blood of suddenly dead pigs (Vela et al., 2006). In addition, *C. xerosis* is mentioned in other veterinary contexts, such as colonization of pig foetuses, which could be detected after abortion (Palacios et al., 2010). Although there are great similarities to the human‐relevant species *C. freneyi* and *C. amycolatum*, harmful infections of *C. xerosis* have been so far found only sporadically in immunosuppressed individuals (Palacios et al., 2010). Nevertheless, data are very limited and precise conclusions about the pathogenicity of the pathogen in pigs and human cannot be made because *C. xerosis* has always been found in association with other pathogens, as in this study.

In porkers barn results revealed that Prevotellaceae is the most prevalent family (24% unique peptides of all identified bacterial peptides) in this bioaerosol (Figure 4) and *Prevotella* being the dominant genus (Supporting Information 2). This bacterial genus is already known to be one of the most abundant bacterial genera in the intestines of pigs and has been found to be highly enriched, especially after weaning from milk and switching to a plant‐based diet (Amat et al., 2020; Chen et al., 2021; Choudhury et al., 2021; Ke et al., 2019). Since commensal *Prevotella copri* is also found in humans in association with arthritis, their role in human health is considered controversially (Drago, 2019; Maeda & Takeda, 2019).

The other dominant phylum in both sow and porker barns are the Firmicutes. However, no dominantly occurring families were detected in either type of barn. Rather, peptides could be detected, which correspond to Clostridiaceae, Lactobacillaceae, Staphylococcaceae, Veillonelaceae and Streptococaceae as well as other bacterial families (Figure 4).

### Functional aspects of the metaproteome of bacteria

In this study, some interesting species among the identified bacteria were summarized in Table 2. These include either those bacteria found with large numbers of their unique peptides in the bioaerosol, or those that can be highlighted due to their potential pathogenicity according to the German Technical Rules for Biological Agents (TRBA 466). To take advantage of their potential pathogenicity, these species must (a) be present in sufficient numbers, (b) maintain their metabolic functions in airborne state, and (c) still be able to attach to, (d) grow on, and (e) penetrate the host (Wilson, 2002).

**TABLE 2:** Selection of interesting species identifications and their classification in TRBA 466.

Bacterial phylum	Bacterial species	TRBA entry	Sows bioaerosol (proportion % of bacteria)	Porkers bioaerosol (proportion % of bacteria)		
Actinobacteria	*Corynebacterium xerosis*	1 t+	3/3 days (6.13%)	3/3 days (0.06%)
	*Corynebacterium freneyi*	2	3/3 days (0.50%)	Na
Bacteroidetes	*Prevotella copri*	1	3/3 days (0.95%)	3/3 days (9.73%)
	*Prevotella* bryantii	2	Na	3/3 days (0.11%)
Firmicutes	*Faecalibacterium prausnitzii*	2	Na	3/3 days (0.24%)
	Megasphaera elsdenii	1 ht	3/3 days (0.14%)	3/3 days (3.09%)
	*Acidaminococcus fermentans*	2 ht	na	3/3 days (0.30%)
	*Turicibacter sanguinis*	1+	3/3 days (0.59%)	na

**FIGURE 5:**
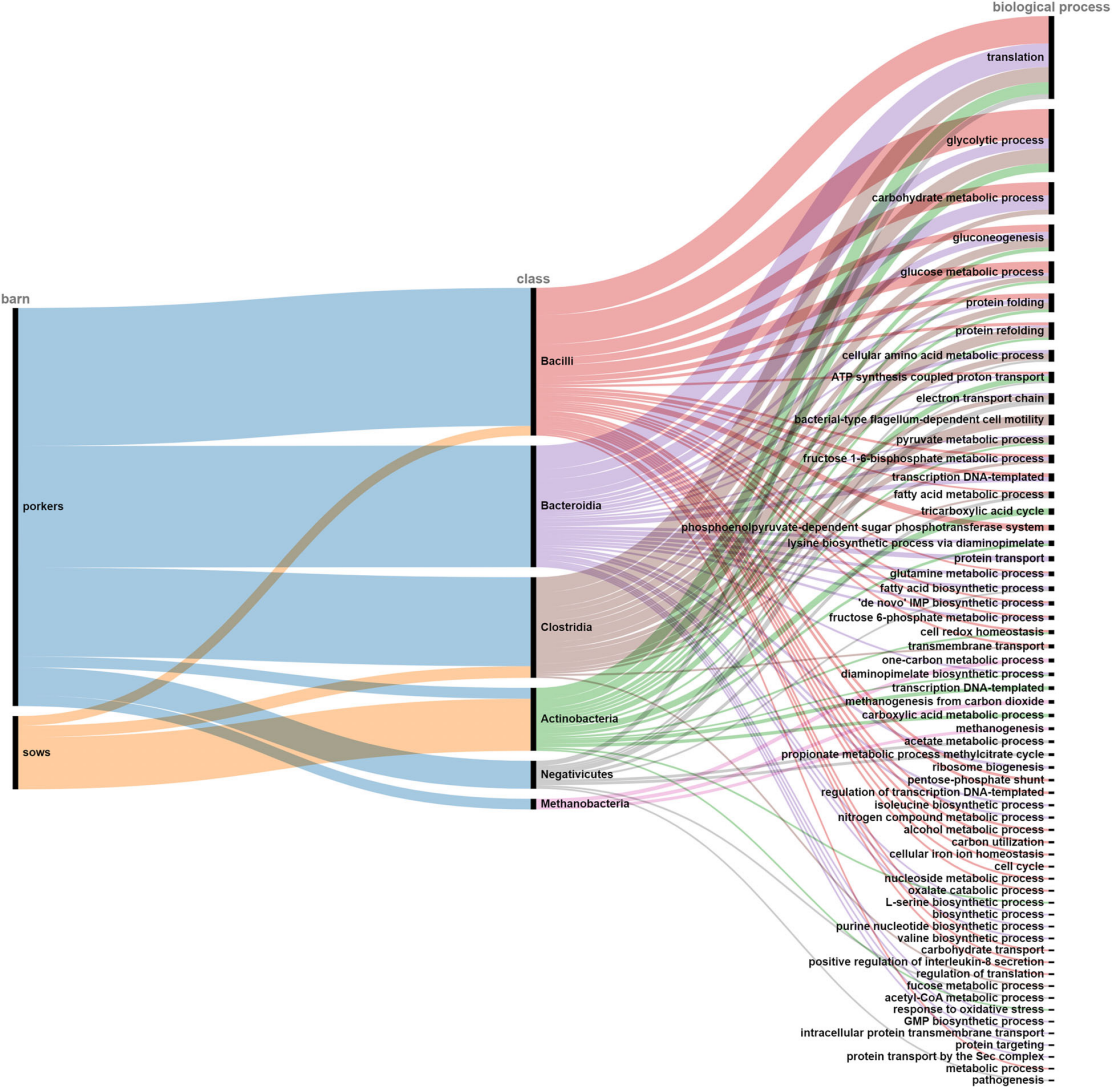
Overview of identified bacterial classes with link to corresponding GO‐term assignments of biological processes.

In this context, just a small number of unique peptides compared to the number of all peptides was found from potentially pathogenic species classified in risk group 2. However, one has to consider that additional peptides could belong to the pathogenic species but are too nonspecific to be matched at the species level and therefore can only be assigned to higher taxonomic levels. However, further efforts are needed to actually quantify these pathogenic species in the bioaerosol and determine the quantitative possibility and physiological capability of infection. Hints for the latter come from examining the protein profile of the airborne microorganisms and highlighting relevant metabolic proteins. Figure 5 provides an overview of identified peptides assigned to specific biological processes using the gene onthology (GO) database. Based on this assignment, it is clear that a large proportion of the peptides belong to the so‐called housekeeping functions of metabolically active bacterial cells and of those proteins that are indispensable for cellular processes. However, it could not be determined whether the microorganisms maintain their metabolism in air. The analysed proteome could be a snapshot of continuously aerosolized cells from the sludge with immediate drying in air.

Furthermore, peptides in porker bioaerosol were allocated to the GO term (biological process) “pathogenicity” (100%). Some peptides can be assigned to *Megasphaera elsdenii* with homologies to a YadA‐like protein (IPR008635) from *Yersinia enterocolitica* (Roggenkamp et al., 2003), or homologies to a superfamily type, the C‐terminal serralysin‐like metalloprotease (IPR011049). This protease type is found in various bacteria, has a largely conserved repeats‐in‐toxin (RTX) domain at its C‐terminus, and is cytotoxic (Park & Ming, 2002; Stella et al., 2017). However, these findings need further investigation.

In addition, results suggest the presence of a peptide of the pore‐forming toxin cytolysin from *Trueperella pyogenes* and a peptide from *Pseudomonas* associated with an RTX calcium‐binding nonapeptide repeat type (IPR001343).

Summarizing, the microbiome of the pigs has an effect of the barn bioaerosols, as already described in previous studies (Kraemer et al., 2018; Yan et al., 2021). We found evidence for a different microbial composition within the sow bioaerosol compared to the porker bioaerosol. It has already been suggested that the main source of bioaerosols may be from swine manure (Kristiansen et al., 2012; Nehmé et al., 2008). Considering the results of Gierse et al. (2020), we conclude that our results show similarities to the results of faeces from young pigs switching to a plant‐based diet. Thus, proteins from faecal microbiota, the pigs themselves and their feed participate in the metaproteomic composition of the bioaerosols. We assume that the manual cleaning operations in the barn by the employees increase the stirring up of faecal microorganisms and other factors into the air, besides continuous bubble bursting in the manure by microbial activity. In addition, splashing of new slurry into the throughs, promotes aerosolization of microorganisms. Thus, reorganization of the manure management, for example, continuous transport or rinsing would probably decrease bioaerosol formation from the manure in the pig barn. Also sufficient ventilation, if possible close to the source of bioaerosol formation would likely decrease bioaerosol formation in the pig barn. Besides the mentioned technical measures for employees also operational measures, for example, proper cleaning protocols with appropriate wearing of personal protective equipment, would also be beneficial to employees and mitigate exposure to bioaerosols.

Occupational diseases in agriculture related to inhalation exposure to fungi and bacteria have already been identified (Nordgren & Charavaryamath, 2018). Nevertheless, it should be noted that there is also evidence of a protective effect of agricultural workers against sensitization by common allergens (Elholm et al., 2013; Riedler et al., 2001).

## CONCLUSION

The high risk of biological contamination in agricultural workplaces, especially in intensive livestock farming, is a major issue in occupational safety and health. However, complex compound bioaerosols can only be analysed and evaluated inadequately so far. Here, we demonstrate that the chosen metaproteomic workflow is a powerful technique for a more comprehensive analysis of bioaerosols in such workplaces.

Metaproteomics can be used to detect both the structure of the microbial community, allowing conclusions to be drawn about the infection potential, and the presence of expressed proteins that have allergenic or toxic potential. It is therefore conceivable that the health‐damaging potential of a bioaerosol could be assessed by metaproteomic studies. Further work needs to be done on detection of virulence factors and in particular on the quantification of airborne allergens and toxins in order to assess the adverse health potential of a bioaerosol by protein‐based assays.

## AUTHOR CONTRIBUTIONS

**Susann Meyer:** Conceptualization (equal); data curation (equal); methodology (lead); visualization (lead); writing – original draft (lead); writing – review and editing (equal). **Nicole Hüttig:** Methodology (equal); writing – review and editing (equal). **Marianne Zenk:** Resources (lead); writing – review and editing (equal). **Udo Jäckel:** Conceptualization (equal); writing – review and editing (equal). **Dierk‐Christoph Pöther:** Conceptualization (equal); data curation (equal); writing – review and editing (equal).

## CONFLICT OF INTEREST STATEMENT

The authors declare no conflict of interest.

## ETHICS STATEMENT

The authors confirm that the ethical guidelines of the journal, as stated in the journal's Author Guidelines, have been followed. Ethics committee approval was not required for the sampling procedure within the study, as no studies were conducted with animal or human participants and only stationary collection systems were used to collect bioaerosol particles from barn air.

## Supporting information


**Data S1** Supporting Information.

**Data S2** Supporting Information.

## Data Availability

Amplicon sequencing data were deposited to the NCBI BioProject Database with accession number PRJNA1004577. The mass spectrometry proteomics data have been deposited to the ProteomeXchange Consortium via the PRIDE partner repository and with the dataset identifier PXD039685.
